# Amino Acid Residue 217 in the Hemagglutinin Glycoprotein Is a Key Mediator of Avian Influenza H7N9 Virus Antigenicity

**DOI:** 10.1128/JVI.01627-18

**Published:** 2018-12-10

**Authors:** Pengxiang Chang, Joshua E. Sealy, Jean-Remy Sadeyen, Munir Iqbal

**Affiliations:** a The Pirbright Institute, Pirbright, United Kingdom; St. Jude Children's Research Hospital

**Keywords:** H7N9 virus, immune escape, antigenic diversification, avian influenza virus, evolution, hemagglutinin glycoprotein

## Abstract

Avian influenza viruses continue to evolve and acquire mutations that facilitate antigenic drift and virulence change. In 2017, low-pathogenicity H7N9 avian influenza viruses evolved to a high-pathogenicity phenotype in China. Comparative antigenic analysis of the low- and high-pathogenicity virus strains showed marked variability. In order to identify residues that may be linked to the antigenic change among the H7N9 viruses, we serially passaged the viruses in the presence of homologous ferret antiserum. Progeny viruses able to overcome the neutralizing capacity of the antiserum were sequenced. The analysis showed that the emergent immune escape viruses contained mutations A125T, A151T, and L217Q in the hemagglutinin (HA) glycoprotein as early as passage 5 and that these mutations persisted until passage 10. The results revealed that a single mutation, L217Q, in the HA of H7N9 virus led to 23- and 8-fold reductions in hemagglutination inhibition (HI) titer with ferret and chicken antisera, respectively. Further analysis showed that this change also contributed to antigenic differences between the low- and high-pathogenicity H7N9 viruses, thus playing a major role in their antigenic diversification. Therefore, evolutionary changes at amino acid position 217 in the H7N9 viruses can serve as a genetic marker for virus antigenic diversity during vaccine seed matching and selection. The *in vitro* immune escape mutant selection method used in this study could also aid in the prediction of emerging antigenic variants in naturally infected or immunized animals.

**IMPORTANCE** Avian influenza H7N9 viruses circulating in poultry and wild birds continue to evolve and acquire important phenotypic changes. Mutations to the virus hemagglutinin (HA) glycoprotein can modulate virus antigenicity and facilitate virus escape from natural or vaccine-induced immunity. The focus of this study was to identify evolutionary markers in the HA of H7N9 that drive escape from antibody-based immunity. To achieve this, we propagated low-pathogenicity H7N9 virus in the presence of polyclonal antiserum derived from ferrets infected with the same strain of virus (homologous antiserum). This selection process was repeated 10 times. The HA gene sequences of viruses recovered after the fifth passage showed that the viruses readily acquired mutations at three different amino acid positions (A125T, A151T, and L217Q). Further functional analysis of these mutations confirmed that the mutation at residue 217 in the HA was responsible for mediating changes to the immunological properties of the H7N9 virus.

## Abstract (precis)

Avian influenza H7N9 viruses circulating in poultry and wild birds continue to evolve and acquire important phenotypic changes. Mutations to the virus hemagglutinin (HA) glycoprotein can modulate virus antigenicity and facilitate virus escape from natural or vaccine-induced immunity. The focus of this study was to identify evolutionary markers in the HA of H7N9 that drive escape from antibody-based immunity. To achieve this, we propagated low-pathogenicity H7N9 virus in the presence of polyclonal antiserum derived from ferrets infected with the same strain of virus (homologous antiserum). This selection process was repeated 10 times. The HA gene sequences of viruses recovered after the fifth passage showed that the viruses readily acquired mutations at three different amino acid positions (A125T, A151T, and L217Q). Further functional analysis of these mutations confirmed that the mutation at residue 217 in the HA was responsible for mediating changes to the immunological properties of the H7N9 virus.

## INTRODUCTION

Low-pathogenicity avian influenza (LPAI) H7N9 virus infection in humans was first reported in China in February 2013. Since then, there have been 1,625 confirmed human H7N9 virus infections and 623 deaths, a case fatality rate of almost 40% (1). There have been five epidemic waves of infection since H7N9 viruses first emerged; high-pathogenicity avian influenza (HPAI) variant H7N9 viruses emerged in wave 5 during 2017 and are currently cocirculating with the LPAI H7N9 viruses. According to phylogenetic analyses, there are two distinct lineages of H7N9 virus established in China, namely, the Yangtze River Delta lineage and the Pearl River Delta lineage; all HA genes of HPAI H7N9 viruses belong to the Yangtze River Delta lineage (2). As in chickens, HPAI H7N9 viruses display higher pathogenicity in mice and ferrets than LPAI H7N9 viruses (3). Antigenic analysis has revealed low or no cross-reactivity between LPAI and HPAI H7N9 virus strains by hemagglutinin inhibition (HI) assay using ferret antiserum (4). Antigenic divergence has also been observed in a mouse vaccination/challenge model (5). These observations describe significant antigenic drift between LPAI and the HPAI H7N9 viruses. However, the molecular determinants that mediate the differential antigenic properties of LPAI and HPAI H7N9 viruses remain elusive.

H7N9 viruses isolated from humans have shown strong binding avidity for the human-like receptor analog α-2,6-sialyllactosamine (6SLN) and equally retained binding avidity for avian-like receptor analog α-2,3-sialyllactosamine (3SLN) (6, 7). This dual receptor binding ability is potentially linked to mutations at HA positions 217 and 177 (the numbering for mature H7 HA is used throughout) (7). In addition to human receptor binding characteristics, human H7N9 isolates often possess several other markers of mammalian adaptation, such as E627K and D701N in the PB2 protein (8). Animal experiments indicate that avian-origin H7N9 viruses can transmit among ferrets without prior adaptation, highlighting their pandemic potential (3).

Propagation of influenza viruses *in vitro* under immune pressure can give rise to antigenic variant viruses. Methods of *in vitro* selection have previously been used to assess the antigenic evolution of influenza viruses such as HPAI H5N1 virus and seasonal influenza viruses (9, 10). The identified escape mutants have also been seen in nature with reciprocal impacts on antigenicity, thus exemplifying the relevance of this approach. In this study, we used *in vitro* selection to identify key escape mutations in the HAs of LPAI H7N9 viruses and further characterized their contribution to antigenic change between the contemporary LPAI and HPAI H7N9 viruses infecting poultry and humans in China.

## RESULTS

### The L217Q mutation is a key mediator of antigenic change in LPAI H7N9 viruses.

**FIG 1:**
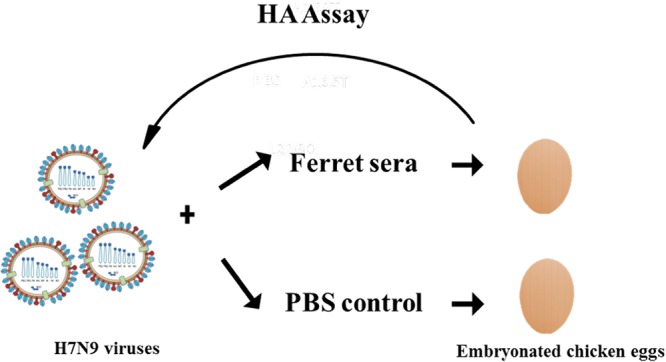
Schematic presentation of *in vitro* antiserum escape mutant selection procedure. Anhui/13 virus was mixed with serially diluted ferret antiserum or PBS as control and inoculated into embryonated chicken eggs. The HA-positive egg allantoic fluid from the highest antiserum concentration was again mixed with antiserum as before and passaged in eggs.

**FIG 2:**
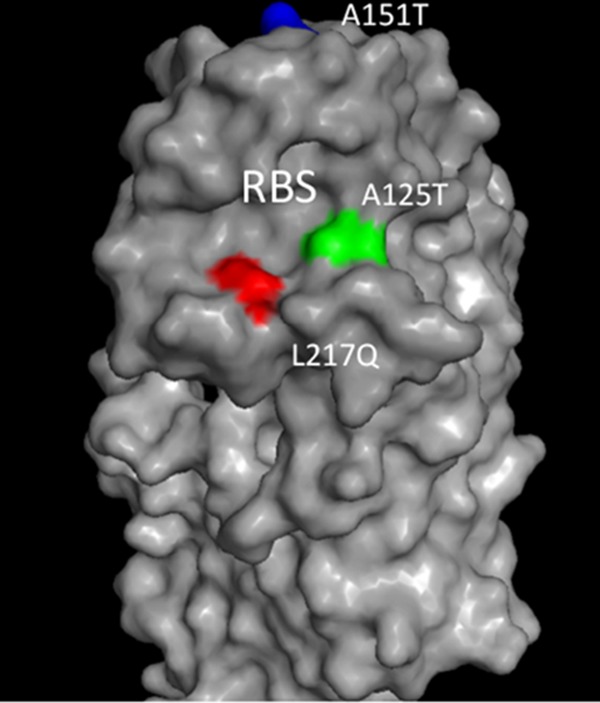
Location of the HA mutation residues based on the HA monomer three-dimensional structure of H7N9 influenza virus (A/Shanghai/1/2013) (Protein Data Bank [PDB] accession no. 4LN3**) (**32**).** RBS, receptor binding site. The mutations A125T, A151T, and L217Q (mature H7 HA numbering) are indicated in green, blue, and red, respectively.

To investigate antigenic change under immune pressure, an *in vitro* selection method was adopted. Anhui/13 virus was mixed with ferret antiserum or phosphate-buffered saline (PBS) as a control and inoculated into embryonated chicken eggs (Fig. 1). The virus-positive egg allantoic fluid from the highest viable antiserum concentration was again mixed with antiserum and passaged in eggs. The progeny viruses showed amino acid mutations including changes from alanine 125 to threonine (A125T), alanine 151 to threonine (A151T), and leucine 217 to glutamine (L217Q) (the numbering for mature H7 HA is used throughout) in the HAs of all 12 selected cDNA clones analyzed as early as passage 5. These identified amino acid substitutions correspond to A135T, A160T, and L226Q by the H3 HA numbering. HA gene sequence analysis of the viruses passaged in eggs with PBS control revealed one clone with a change from glycine 209 to glutamate (G209E), two clones with the A180E change, four clones with the G189E change, two clones with the double mutation of asparagine 149 to aspartate (N149D) and valine 86 to A (V86A), and three clones with no identified mutations. The comparative analysis of the HA genes of viruses passaged in the presence of ferret serum and the PBS control indicated that the A125T, A151T, and L217Q mutations arose only in viruses propagated with the immune antiserum and not through egg adaption. We carried out the selection procedure for a further 5 passages; no further mutations in addition to A125T, A151T, and L217Q were detected. Both the A125T and L217Q mutations are located within the receptor binding site (RBS) (Fig. 2), while A151T is located in the distal region of HA1. Both the A125T and A151T substitutions introduced N-linked glycosylation motif (N-X-T/S), and changes in the glycosylation pattern in the HA can interfere with antibody binding (11). To investigate the antigenic change of the escape mutant viruses and further explore the contributions of each mutation, individual substitutions and substitutions in different combinations were reintroduced into the HA of Anhui/13 by site-directed mutagenesis, and mutants were reconstituted through virus rescue as LPAI H7N9:PR8 2:6 recombinants (Table 1). Compared with the Anhui/13 virus, the serum escape mutant (A125T+A151T+L217Q) showed an 11-fold reduction in hemagglutinin inhibition (HI) titer to the ferret homologous antiserum and a 7-fold reduction in HI titer to the chicken homologous antiserum. This slight variability in reduction of HI titers between the ferret and chicken antisera could be due to the different antibody repertoires that may exist in ferrets and chickens in response to H7N9 avian influenza virus infection. The single mutation A125T or A151T resulted in a <4-fold reduction in HI titer to ferret and chicken antisera, respectively (Table 1). However, the A125T+A151T double mutant virus displayed an 8-fold reduction in HI titer to ferret antiserum and a 4-fold reduction in HI titer to the chicken antiserum, indicating that these two substitutions had a cumulative antigenic effect. Surprisingly, the L217Q mutation alone yielded a 23-fold reduction in HI titer to the ferret antiserum and an 8-fold reduction in HI titer to the chicken antiserum. Incorporation of the additional mutation A125T along with L217Q did not dramatically affect the HI titers observed with L217Q mutation alone. However, the mutant L217Q virus containing the A151T mutation showed a compromise in the antigenic change caused by the L217Q substitution. This double mutant showed 8- and 5-fold reductions in HI titers to ferret and chicken antisera, respectively, compared to Anhui/13 (L217) (Table 1). Overall, these results indicated that the L217Q mutation is a key mediator of antigenic change in LPAI H7N9 viruses.

**TABLE 1:** Contributions of serum escape mutations to the antigenic change of H7N9 viruses

Strain and mutation(s)	Ferret serum^*a*^		Chicken serum^*a*^	
	Fold reduction in HI titer^*b*^	HI titer^*c*^	Fold reduction in HI titer^*b*^	HI titer^*c*^
Anhui/13 (L217)				
Anhui/13		181		128
A125T+A151T+L217Q	11	16	7	19
A125T+A151T	8	23	4	32
A125T+L217Q	27	7	8	16
A151T+L217Q	8	23	5	27
A125T	3	64	2	76
A151T	1	152	2	64
L217Q	23	8	8	16
HK125/17 (L217)				
HK125/17	4	45	3	45
L217Q	23	8	16	8

a Chicken and ferret antisera used in these assays were raised against Anhui/13 virus. The results shown are representative of three experimental repeats.

b Observed fold reduction in HI titers of the indicated viruses compared with Anhui/13.

c HI titers of the indicated mutant viruses using antiserum raised against Anhui/13.

To further confirm the impact of the leucine-to-glutamine change at residue 217 on the antigenicity of H7N9 viruses, we reconstituted another recombinant virus that contained the HA and neuraminidase (NA) genes from LPAI H7N9 virus (A/Hong Kong/125/2017, isolated during epidemic wave 5) and internal gene from PR8 virus (referred to as HK125/17). Substitution L217Q was introduced into the HA of HK125/17 by site-directed mutagenesis. The HK125/17 mutant virus with the L217Q substitution showed 23- and 16-fold reductions of HI titers with ferret and chicken antisera raised against Anhui/13, respectively. However, the HK125/17 virus that contains L217, similar to the Anhui/13 virus, also showed 4- and 3-fold reductions in HI titers to Anhui/13 virus-specific ferret and chicken antisera, respectively, compared with Anhui/13 virus (Table 1). This suggests that although substitution L217Q in the HA induces a large antigenic change, other amino acid differences in HA between HK125/17 and Anhui/13 (A112T, S118N, and A125V) may also to a minor extent contribute to antigenic variability among the different strains of H7N9 viruses.

### Mutations at amino acid residues 125, 151, and 217 have emerged in nature.

Polymorphism analysis was performed on HA sequences, retrieved from the Global Initiative on Sharing All Influenza Data (GISAID) database, from the past five H7N9 epidemic waves in China (12). All three serum escape mutations, A125T, A151T, and L217Q, were found in naturally occurring H7N9 isolates (Table 2). The A125T substitution was found only in wave 2. Notably, the A125V substitution first emerged in wave 2 and its detection among H7N9 isolates increased from 6.9% in wave 2 to 95% in wave 5. The percentage of isolates containing A151 did not dramatically change over the past 5 epidemic waves, although detection of T151 did increase from none in wave 4 to 1.4% in wave 5. The amino acid identity at residue 217 has greatly fluctuated since 2013; it decreased from 13.5% Q217 in wave 1 to less than 4% in waves 2, 3, and 4 and then increased again to 14.3% in wave 5 (Table 2). To assess the linkage of the identified mutations to risk of human adaptation, we compared the frequencies of those substitutions in the HAs of H7N9 viruses isolated from human and avian hosts. The rates of both the A125T and A151T mutations appeared to be higher in human than in avian virus isolates, indicating that these two mutations that carry glycosylation motifs may play some adaptive role for humans, though the prevalence rates of both mutations are less than 1% (Table 3). The L217Q mutation prevailed more often in the avian host (7.22%) than in the human host (3.98%) (Table 3). This indicated that glutamine at position 217 likely has a fitness advantage over leucine in the avian host.

**TABLE 2:** Polymorphism analysis of HA residue 125, 151, and 217 in the five H7N9 epidemic waves

H7N9 epidemic wave	% of sequences^*a*^ with the indicated amino acid residue at position:									
	125				151			217		
	A	T	V	Other	A	T	Other	Q	L	Other
1	99.0	0	0	1.0	99.5	0	0.5	13.5	84.1	2.4
2	91.7	0.9	6.9	0.6	98.9	0.6	0.6	1.6	97.4	1.0
3	42.0	0	55.6	2.3	99.2	0.4	0.4	3.9	87.6	8.5
4	5.9	0	92.9	1.1	98.8	0	1.2	1.2	97.6	1.2
5	4.8	0	95.0	0.2	97.6	1.4	1.1	14.3	84.1	1.7

a All available H7N9 virus sequences were retrieved from the Global Initiative on Sharing All Influenza Data (GISAID) from 1 January 2013 to 1 September 2017 in China. Wave 1 is from 1 January 2013 to 1 September 2013 (*n* = 208), wave 2 is from 1 September 2013 to 1 September 2014 (*n* = 700), wave 3 is from 1 September 2014 to 1 September 2015 (*n* = 258), wave 4 is from 1 September 2015 to 1 September 2016 (*n* = 85), and wave 5 is from 1 September 2016 to 1 September 2017 (*n* = 666) (12).

**TABLE 3:** Frequencies of A125T, A151T, and L217Q substitutions in HAs of H7N9 avian influenza viruses isolated from human and avian hosts

Species	% (no./total) of sequences^*a*^ with substitution:		
	A125T	A151T	L217Q
Human	0.40 (5/1,257)	0.88 (11/1,257)	3.98 (50/1,257)
Avian	0.15 (1/679)	0.29 (2/679)	7.22 (49/679)

a All available H7N9 virus sequences were retrieved from GISAID from 1 January 2013 to 1 September 2017 in China (*n* = 1,936).

### Amino acid residue Q217 is a critical residue that accounts for the weak cross-reactivity between LPAI and HPAI H7N9 viruses.

**FIG 3:**
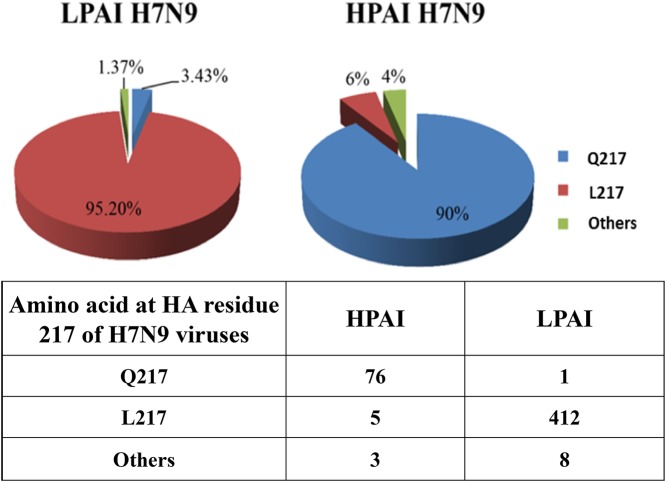
Analysis of H7N9 HA residue 217 polymorphism during epidemic wave 5 in China. Amino acid identity is shown by color. All available HPAI (*n* = 83) and LPAI (*n* = 583) H7N9 virus sequences were retrieved from GISAID database during epidemic wave 5 in China (from 1 September 2016 to 1 September 2017). The table shows the numbers of LPAI and HPAI H7N9 viruses that carry glutamine (Q), leucine (L), or other amino acid residues at position 217.

The available HA sequences in the public databases showed that 90% of the HPAI H7N9 viruses contained residue Q217 in wave 5. Conversely, the HAs of more than 95% of the LPAI H7N9 viruses had L217, and only 3.43% of the sequenced HAs of LPAI viruses contained Q217 (Fig. 3). The dramatic difference in the amino acid at residue 217 between LPAI and HPAI H7N9 viruses prompted us to look into whether the Q217 motif was responsible for the low cross-reactivity of LPAI H7N9 antiserum with HPAI H7N9 viruses as seen in ferret and mouse models (4, 5). Both the HPAI H7N9 A/Guangdong/17SF003/2016 and A/Guangdong/17SF006/2017 virus strains, which harbor Q217 in their HAs, showed low or no reactivity with antiserum to the LPAI H7N9 Anhui/13 virus strain, which contains L217 (4). To investigate the contribution of Q217 in the antigenic cross-reactivity, recombinant H7N9 virus containing HA from HPAI H7N9 SF003 and NA from Anhui/13 was generated in the PR8 genetic background (referred to as SF003-HA). The Q217L substitution was introduced into SF003-HA by site-directed mutagenesis. Consistent with previous reports, SF003-HA had 23- and 6-fold reductions in HI titers to ferret and chicken antisera, respectively, compared with Anhui/13 (Table 4). As expected, compared with SF003-HA (Q217), the mutant SF003-HA with the Q217L substitution showed 8- and 3-fold increases in HI titers to ferret and chicken antisera, respectively. However, in comparison to the LPAI Anhui/13, mutant SF003-HA/Q217L virus still displayed 3- and 2-fold lower HI titers to ferret and chicken antisera, respectively. Further comparison of HA sequences between Anhui/13 and SF003-HA revealed that in addition to the L217Q mutation, there are 6 other substitutions in the HA1 of SF003-HA: changes of isoleucine 38 to threonine (I38T), alanine 112 to proline (A112P), serine 118 to asparagine (S118N), alanine 125 to valine (A125V), lysine 164 to glutamate (K164E), and glycine 261 to arginine (G261R). Among all of these substitutions, the prevalence of A125V in the HA of the mutant virus showed a sharp increase in epidemic wave 5 (Table 2) and has been shown to have a cumulative antigenic effect (13). To investigate the contribution of V125 in the antigenic cross-reactivity, V125A and V125A+Q217L substitutions were introduced into SF003-HA by site-directed mutagenesis. The V125A+Q217L double mutations in SF003-HA completely abolished the antigenic difference between the Anhui/13 and SF003-HA viruses (Table 4). Surprisingly, V125A alone did not affect the antigenicity of SF003-HA, indicating the cumulative antigenic effect of V125 and Q217 in SF003-HA. To further confirm the findings in the SF003-HA background, we introduced A125V and A125V+L217Q double mutations into Anhui/13. Consistent with the observed results with SF003-HA, the A125V substitution did not affect the antigenicity of Anhui/13. However, reconstitution of Anhui/13 virus that carried L217Q+A125V did not showed a marked difference in HI titers compared with those of virus that contained L217Q alone, indicating that the cumulative antigenic effect of L217Q+A125V might be linked to other amino acid differences between HA1 (I38T, A112P, S118N, K164E, and G261R) of the Anhui/13 and SH003-HA viruses.

**TABLE 4:** Contributions of residue 125 and 217 mutations to H7N9 virus HA antigenic cross-reactivity

Strain and mutation(s)	Ferret serum^*a*^		Chicken serum^*a*^	
	Fold reduction in HI titer^*b*^	HI titer^*c*^	Fold reduction in HI titer^*b*^	HI titer^*c*^
Anhui/13 (L217)		181		128
SF003-HA (Q217)	23	8	6	23
SF003-HA				
Q217L	3	64	2	64
Q217L+V125A	0	181	0	128
V125A	23	8	6	23
Anhui/13				
A125V	0	181	0	128
L217Q	23	8	8	16
A125V+L217Q	23	8	8	16

a Chicken and ferret antisera used in these assays were raised against Anhui/13 virus. The results shown are representative of three experimental repeats.

b Observed fold reduction in HI titers of the indicated mutant viruses compared with Anhui/13.

c HI titers of the indicated mutant viruses using antiserum raised against Anhui/13.

## DISCUSSION

H7N9 avian influenza viruses continue to evolve and pose a huge risk to the poultry industry as well as to human health. In this study, we used an *in vitro* antiserum escape mutant selection method to the study antigenic change of these viruses under immune pressure. We demonstrated that residue 217 is a major antigenic epitope for H7N9 avian influenza viruses and that the L217Q substitution resulted in large reductions in both ferret and chicken antiserum binding. Additionally, we showed that residues Q217 and V125 in HA are responsible for the weak cross-reactivity between LPAI and HPAI H7N9 viruses.

Residue 217 plays a prominent role in the receptor binding specificity of HA. In several subtypes L217 is associated with binding to human-like receptors, while Q217 at this position is associated with binding to avian-like receptors (14). However, its role in the H7N9 viruses is intriguing. LPAI H7N9 virus (Anhui/13) maintains its dual receptor binding capacity for both avian and human hosts despite the L217Q substitution (15). In this study, we showed that Q217 was more frequently found in avian than in human virus isolates. As a result, it will be interesting to examine the differences in receptor binding when the L217Q substitution is introduced into the current prevailing virus strains. In addition to the receptor binding switch, L217Q together with an N149D or G209E substitution was also found in the H7N9 virus egg adaptation mutant (16). Recently, L217 was identified as an antigenic epitope for LPAI H7N9 viruses using a human monoclonal antibody (17). However, its contribution to the antigenic change of H7N9 virus is not well understood. Here, we showed that the L217Q substitution drove a major antigenic change in H7N9 viruses under immune pressure. In epidemic wave 5, both LPAI and HPAI H7N9 viruses are found cocirculating; however, the LPAI H7N9 strains are still predominant in China, and the majority of these LPAI H7N9 strains contain L217. Here, we showed that the L217Q substitution in the HA of HK125/17, a virus isolate from epidemic wave 5, resulted in large reductions in HI titers to ferret and chicken antisera in comparison to those with Anhui/13. It is therefore imperative to closely monitor the polymorphism at residue 217 to assess the need to update vaccine seed strains.

In addition to the L217Q substitution, A125T and A151T double mutations resulted in modest reductions in HI titers to ferret and chicken antisera. Both of these substitutions gain a potential N-linked glycosylation motif (N-X-T/S) and may result in the glycosylation of HA. Residue 125 is in adjacent to the RBS, and glycosylation at this site in HPAI H7N7 virus has been shown to increase binding avidity for avian-type receptor analogs and enhance replication efficiency in MDCK cells (18, 19). Likewise, the glycosylation of H7N9 HA by the A125T substitution has been shown to favor production of HA pseudoviruses (20). It is noteworthy that the A125T substitution has been found to emerge in the HA of an H7N9 virus isolated from a contact ferret during a transmission experiment (21). Here, we showed that A125T and A151T were found more often in human than in poultry isolates, although the sample size is small. Therefore, their impact on glycan binding warrants further investigation. The A151T substitution has also previously introduced glycosylation in H5N1, resulting in reduced virus binding toward human-like receptors (22). However, validation of the potential glycosylation in the mutant A151T in H7N9 viruses still needs to be performed. Although A125T and A151T substitutions were identified as egg adaptation mutations, the A125T+A151T double mutation was not found in the egg-amplified wild-type H7N9 viruses (23). N-linked glycosylation sites in the globular head domain can sterically block the binding of antibody to multiple sites, which is one of the most efficient ways for influenza virus to escape from antibody-mediated neutralization (24). The mutations at residues 125 and 151 have been known to drive the variability in glycosylation in the HA, and these sites have been implicated in influencing antigenic diversity in influenza viruses (25–27). Therefore, we speculate that these two mutations at residues 125 and 151 arose due to the presence of antiserum, forcing H7N9 viruses to escape from immune pressure.

The government of China implemented a mass vaccination program of poultry against H7N9 in 2017, with a vaccine that contains the HA antigen derived from an Anhui/13-like candidate vaccine virus (CVV). Recent analysis shows that the HA antigens of contemporary field viruses isolated in 2017 have undergone marked antigenic change; in particular, the newly emergent HPAI H7N9 viruses of the fifth wave showed relatively low cross-reactivity with the CVV strain (Anhui/13) (28). It is therefore critical to keep the vaccine updated for vaccination to remain effective. Due to the antigenic shift, the World Health Organization (WHO) recommended HK125/17 and SF003 as LPAI and HPAI H7N9 CVV strains, respectively (28). Compared with the 2013 CVV strain Anhui/13, HK125/17 has A112T, S118N, and A125V substitutions in the HA, while SF003 has 7 substitutions in the HA: I38T, A112P, S118N, A125V, K164E, L217Q, and G261R. Here, we demonstrated for the first time that V125 and Q217 alone are responsible for the marked low cross-reactivity between the LPAI and HPAI CVV strains of H7N9 viruses, whereas the antiserum produced against HPAI H7N9 virus (SF003) demonstrated strong cross-reactivity against both the LPAI and HPAI H7N9 viruses (28). From these data, it can be proposed that the CVV strains that are more similar to HPAI SF003 virus have greater antigenic cross-reactivity for both contemporary HPAI and LPAI H7N9 viruses infecting poultry and humans in China.

In summary, A125T, A151T, and L217Q mutations were identified in H7N9 HA using *in vitro* polyclonal antiserum escape mutant selection. Of these three mutations, L217Q was found to be a key mediator of LPAI H7N9 antigenic variation. The Q217 and V125 motif in the HPAI H7N9 virus accounted for the reduced cross-reactivity with LPAI H7N9 antiserum. Moreover, the A125T and A151T double mutations resulted in an 8-fold reduction in HI titer to ferret antiserum. These findings have important implications for CVV strain selection, and the mutations identified here should be monitored closely to assess the virus pandemic potential. Of note, all three mutations (A125T, A151T, and L217Q) identified in the HA of H7N9 immune escape mutants have appeared in nature, indicating that *in vitro* antiserum escape mutant selection is a feasible and powerful tool to predict the antigenic change of influenza viruses.

## MATERIALS AND METHODS

### Ethics statement.

All animal studies and procedures were carried out in strict accordance with the guidance and regulations of European and United Kingdom Home Office regulations under project license number P68D44CF4. As part of this process, the work has undergone scrutiny and approval by the Animal Welfare Ethical Review Board (AWERB) at the Pirbright Institute.

### Viruses and cells.

Recombinant avian influenza H7N9 viruses containing HA and NA from LPAI H7N9 virus (A/Anhui/1/2013) (referred to as Anhui/13), HA and NA from LPAI H7N9 virus (A/Hong Kong/125/2017) (referred to as HK125/17), or HA from HPAI H7N9 virus (A/Guangdong/17SF003/2016) and NA from Anhui/13 (referred to as SF003-HA) were generated in the genetic background of A/Puerto Rico/8/34 (H1N1) (PR8) as previously described (29). The DNA sequences for the HA and NA of H7N9 viruses are derived from the Global Initiative on Sharing All Influenza Data (GISAID). The GISAID accession numbers for the HA of Anhui/13, HA of HK125/17, HA of SF003-HA, NA of Anhui/13, and NA of HK125/17 are EPI439507, EPI977395, EPI439509, EPI919607, and EPI977394, respectively. These HAs and NAs were synthesized by GeneArt (Thermo Fisher Scientific) and then subcloned into PHW2000 plasmids. The polybasic amino acids motif “KRTA” was removed from HA of HPAI H7N9 (A/Guangdong/17SF003/2016) virus. The rescued viruses were propagated in 10-day-old embryonated chicken eggs, and virus stocks were kept at −80°C. To guarantee the genetic purity of all the escape mutants used for downstream antigenic analysis, the identified amino acid substitutions in HA were reintroduced into the reverse genetics plasmids by site-directed mutagenesis. Primers used for the site-specific mutations are listed in Table 5. Madin-Darby canine kidney (MDCK) and human embryonic kidney (HEK) 293T cells (ATCC) were maintained in Dulbecco’s modified Eagle’s medium (DMEM) (Gibco) supplemented with 10% fetal calf sera (FCS) (Gibco), 100 U/ml penicillin, and 100 μg/ml streptomycin (Gibco) at 37°C under a 5% CO_2_ atmosphere.

**TABLE 5:** Primer sequences for H7N9 HA amplification and site-specific mutations

Primer	Sequence (5′→3′)^*a*^
H7N9 HA-Forward	AGTAGAAACAAGGGTGTTTT
H7N9 HA-Reverse	TTCACAACCACTCAAGATGGA
A125T of HA (Anhui/13)-Forward	GAATAAGAACTAATGGA ACAACCAGTGCATGTAGG
A125T of HA (Anhui/13)-Reverse	CCTACATGCACTGGT TGTTCCATTAGTTCTTATTC
A125V of HA (Anhui/13)-Forward	GAATAAGAACTAATGGA GTGACCAGTGCATGTAGGAG
A125V of HA (Anhui/13)-Reverse	CTCCTACATGCACTGGT CACTCCATTAGTTCTTATTC
A151T of HA (Anhui/13)-Forward	CAAACACAGATAATGCT ACATTCCCGCAGATGAC
A151T of HA (Anhui/13)-Reverse	GTCATCTGCGGGAA TGTAGCATTATCTGTGTTTG
L217Q of HA (Anhui/13)-Forward	CGAGACCACAAGTTAATGGT CAATCTGGAAGAATTGACTTTC
L217Q of HA (Anhui/13)-Reverse	GAAAGTCAATTCTTCCAGA TTGACCATTAACTTGTGGTCTCG
Q217L of HA (SF003-HA)-Forward	GACCACAAGTTAATGGT CTATCTGGAAGAATTGAC
Q217L of HA (SF003-HA)-Forward	GTCAATTCTTCCAGA TAGACCATTAACTTGTGGTC
V125A of HA (SF003-HA)-Forward	GAATAAGAACTAATGGG GCAACCAGTGCATGTAGGAG
V125A of HA (SF003-HA)-Forward	CTCCTACATGCACTGGT TGCCCCATTAGTTCTTATTC

a The nucleotide sequence for amino acid mutation indicated in the primer name is underlined.

### Serological testing.

Postinfection ferret antiserum raised against LPAI H7N9 (A/Anhui/1/2013) virus was kindly provided by John McCauley (The Francis Crick Institute, UK). Reference chicken antiserum was raised by immunization with inactivated LPAI A/Anhui/1/2013 H7N9 virus (purchased from the Animal and Plant Health Agency, UK). The hemagglutination titration (HA) and HI assays were performed using 1% chicken blood cells as described in the World Health Organization (WHO) animal influenza training manual (30).

### Selection of serum escape mutant.

Fifty microliters (10^5^ PFU) of Anhui/13 virus was mixed with 50 µl (180 HI units) of serially diluted (10-fold dilution) ferret antiserum or phosphate-buffered saline (PBS) for controls. Virus-antiserum mixtures at various antiserum dilutions were incubated at 37°C for 1 h before inoculation into 10-day-old embryonated chicken eggs for 48 h. Egg allantoic fluid was harvested after 48 h, and the presence of virus was confirmed by HA assay. Allantoic fluid which was positive for virus and from the highest viable concentration of ferret antiserum was again mixed with ferret antiserum as before and passaged in embryonated chicken eggs. This process was repeated for 10 consecutive passages in eggs in the presence of ferret antiserum.

### Sequencing analysis.

Viral RNA was extracted using the QIAamp viral RNA minikit (Qiagen) and reverse transcribed to cDNA with the Verso cDNA synthesis kit (Thermo Scientific) with the universal flu primer (sequence, AGCAAAAGCAGG). The HAs were PCR amplified using PfuUltra high-fidelity DNA polymerase (Agilent) with HA-specific primers (Table 5). The PCR products were then ligated into the pCR-Blunt vector according to the manufacturer’s instructions (Thermo Fisher Scientific). The resultant pCR-Blunt plasmids were Sanger sequenced (SourceBioscience).

### Bioinformatic analysis of amino acid distributions and structures.

All H7N9 HA amino acid sequences were downloaded from GISAID and aligned using MEGA version 6.0 (31). The structural image was generated and rendered in PyMOL (Schrödinger).
